# Ectopic T Cell Receptor-α Locus Control Region Activity in B Cells Is Suppressed by Direct Linkage to Two Flanking Genes at Once

**DOI:** 10.1371/journal.pone.0015527

**Published:** 2010-11-22

**Authors:** Stefan Knirr, Janette Gomos-Klein, Blanca E. Andino, Faith Harrow, Karl F. Erhard, Damian Kovalovsky, Derek B. Sant'Angelo, Benjamin D. Ortiz

**Affiliations:** 1 Department of Biological Sciences, City University of New York, Hunter College, New York, New York, United States of America; 2 Division of Immunology, Memorial Sloan-Kettering Cancer Center, New York, New York, United States of America; New York University, United States of America

## Abstract

The molecular mechanisms regulating the activity of the TCRα gene are required for the production of the circulating T cell repertoire. Elements of the mouse TCRα locus control region (LCR) play a role in these processes. We previously reported that TCRα LCR DNA supports a gene expression pattern that mimics proper thymus-stage, TCRα gene-like developmental regulation. It also produces transcription of linked reporter genes in peripheral T cells. However, TCRα LCR-driven transgenes display ectopic transcription in B cells in multiple reporter gene systems. The reasons for this important deviation from the normal TCRα gene regulation pattern are unclear. In its natural locus, two genes flank the TCRα LCR, TCRα (upstream) and Dad1 (downstream). We investigated the significance of this gene arrangement to TCRα LCR activity by examining transgenic mice bearing a construct where the LCR was flanked by two separate reporter genes. Surprisingly, the presence of a second, distinct, reporter gene downstream of the LCR virtually eliminated the ectopic B cell expression of the upstream reporter observed in earlier studies. Downstream reporter gene activity was unaffected by the presence of a second gene upstream of the LCR. Our findings indicate that a gene arrangement in which the TCRα LCR is flanked by two distinct transcription units helps to restrict its activity, selectively, on its 5′-flanking gene, the natural TCRα gene position with respect to the LCR. Consistent with these findings, a TCRα/Dad1 locus bacterial artificial chromosome dual-reporter construct did not display the ectopic upstream (TCRα) reporter expression in B cells previously reported for single TCRα transgenes.

## Introduction

The molecular mechanisms resulting in T cell-lineage specific gene expression are the subject of much investigation. These studies focus on defining the cis-acting DNA sequences governing the expression of T cell-specifically expressed gene loci, as well as the targets, regulation and activity of a small number of T-lineage biased transcription factors. The picture emerging from these efforts is not a simple one, as the set of transcription factors induced during the stages of T cell commitment are generally not T lineage-specific [1]. Furthermore, *in vivo*, transgenic studies of gene regulatory DNA from archetypal T lineage-specifically expressed gene loci, such as those encoding T cell receptor (TCR)-α and TCR-β chain proteins, report ectopic activity of these cis-acting elements in B cells [2]–[4]. Thus, it is apparent that achieving a more complete understanding of the T-cell gene expression program will require consideration of a wider range of regulatory interactions at gene loci whose products help define the T lineage.

The mouse TCRα gene exists in a complex multi-gene locus on mouse chromosome 14. It is subjected to multiple levels of regulation to produce a specific order and pattern of gene rearrangements and transcription [5]–[7]. This locus contains three differentially expressed genes [TCRα, TCRδ and defender against death (Dad)-1] [8]. This gene arrangement is evolutionarily conserved [9] and juxtaposes the tightly regulated, T cell specific TCR genes with the widely expressed Dad1 gene. The latter encodes a protein with anti-apoptosis function [10] that is required for early embryonic development [11]–[13]. The conservation of this curious juxtaposition raises questions of its potential functional significance in the regulation of these genes in T cells, where both TCRα and Dad1 are highly active, and in B cells, where Dad1 is active, but TCRα is not. In between these two genes lies a stretch of non-coding DNA that supports locus control region (LCR) function [14]. An LCR is distinguished from other cis-acting gene regulatory elements by its ability to function with predictable activity levels and patterns independently of its site of integration in the genome of transgenic mice [15]. The TCRα LCR has been amply demonstrated to confer high-level, lymphoid organ-specific, copy number-related, integration site-independent expression upon linked transgenes in mice [4], [14], [16], [17]. Because TCRα LCR DNA contains elements supporting both T cell specific [17] and widely-active [16], [18], [19] gene regulatory function, we initially hypothesized that it may play a role in regulating and/or coordinating the distinct expression patterns of both the TCRα and Dad1 genes flanking it.

We previously reported that TCRα LCR activity faithfully mimics that of the endogenous TCRα gene during the thymus stages of T cell development including activation at the CD4^neg^CD8^neg^CD25^neg^CD44^neg^ (DN4) thymocyte stage [4]. However, LCR DNA alone was unable to support certain aspects of TCRα gene regulation in the periphery. As alluded to above, significant and reproducible ectopic LCR activity was seen in B cells. These data were generated using single-reporter genes linked, in the natural TCRα gene position, 5′ of the LCR DNA [4]. Therefore, in this system, the LCR lacks several prominent features of its endogenous milieu, including the presence of a gene downstream of it, as well as the specific DNA sequences of the wider TCRα and Dad1 gene loci. We hypothesized that some, or all, of these features provided crucial cis-regulatory information necessary for proper peripheral regulation of TCRα gene expression and proper modulation of TCRα LCR function.

To broaden our perspective on the possible roles of the TCRα LCR in the regulation of the genes that flank it, we created LCR-driven transgenic constructs that contain two separate reporter genes flanking the LCR. One is heterologous and features the human CD2 reporter gene previously studied [20] in the TCRα position with respect to the LCR, and an HLA-B7 reporter fragment linked 3′ to the LCR in the normal Dad1 position [21]. The second is a TCRα/Dad1 bacterial artificial chromosome (BAC) construction in which separate Vα and Dad1 promoter driven reporter genes flank the LCR DNA in its natural context of DNA sequence spanning from the Cα exons through the entire Dad1 genomic locus. Among the significant findings we report here is that neither of the 5′-flanking genes in these “two-gene” reporter systems displayed the ectopic B cell expression that was observed from “single-gene” Vα promoter-driven [2], [3] and heterologous promoter driven [4] reporter constructs studied previously. These data are consistent with a model in which flanking the TCRα LCR with two distinct, active genes results in a T cell restriction to its activity on the upstream-linked gene. This unexpected inter-gene cis-regulatory interaction may be an important component of the mechanisms generating T-lineage specificity of TCRα gene activity.

## Results

### TCRα LCR-driven single-reporter and dual-reporter transgenes

**Figure 1:**
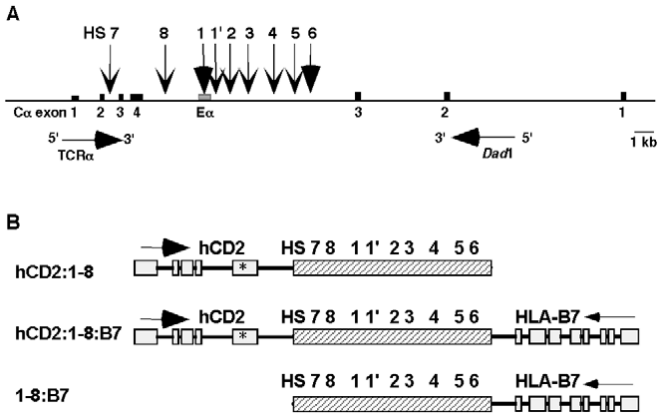
The genomic locus of the TCRα LCR and transgene constructs. (**A**) Scale diagram of the TCRα/Dad1 genomic locus containing the TCRα LCR. (**B**) Diagrams (not drawn to scale) of the three heterologous TCRα LCR reporter transgenes used in these studies. Note that the hCD2 transgene is in the position of the TCRα gene with respect to the LCR sequences. In contrast, the HLA-B7 reporter gene is in the position of the Dad1 gene. Vertical arrows and numbers indicate the nine identified DNase I hypersensitive sites (HS) of the LCR. Horizontal arrows indicate the transcription orientation of the genes depicted. Solid boxes indicate exons. The asterisk denotes the placement of a premature stop codon.

In its native locus, two genes flank the TCRα LCR in the genome (Fig. 1A). To mimic this gene arrangement, we created reporter transgene constructs where the LCR was linked to a single reporter gene on either its 5′- or 3′-end. We also created a “dual-reporter” transgene in which the LCR is flanked by the two different reporter genes at once (Fig. 1B). A human CD2 genomic fragment occupies the normal position of the TCRα gene, linked to the 5′ end of DNase I hypersensitive sites (HS) 1-8 of the LCR. Linked to the 3′-end of the LCR, in the Dad1 position, is an HLA-B7 genomic fragment. The two single-reporter transgenes examined here were named hCD2:1-8 and 1-8:B7. The dual-reporter transgene was named hCD2:1-8:B7. We previously reported the activity of the hCD2-1:8 transgene [4] which encodes a truncated hCD2 protein missing the signaling portion of its cytoplasmic tail [20]. The HLA-B7 reporter has been used previously by others as a transgenic reporter gene [21], [22]. It was chosen as our 3′ reporter gene for this system because it encodes a Class I antigen presentation molecule that, like the Dad1 gene, can be expressed by most cell types. Both gene fragments used are devoid of LCR activity and, thus, are very poorly expressed on their own [21], [23]. Therefore they are suitable reporters of TCRα LCR activity. With these constructs, we initially aimed to test if the TCRα LCR was capable of conferring its powerful regulatory function upon two unrelated genes flanking it simultaneously. Because the TCRα LCR's natural gene neighbors have radically different expression patterns, we also sought to determine the significance of gene position (with respect to the LCR) to the expression pattern of the reporter genes. Nine independent lines of transgenic mice, three for each construct, were created by pronuclear microinjection to enable an *in vivo* examination of these questions.

### The TCRα LCR supports high-level, integration-site independent expression of two simultaneously flanking genes

**Figure 2:**
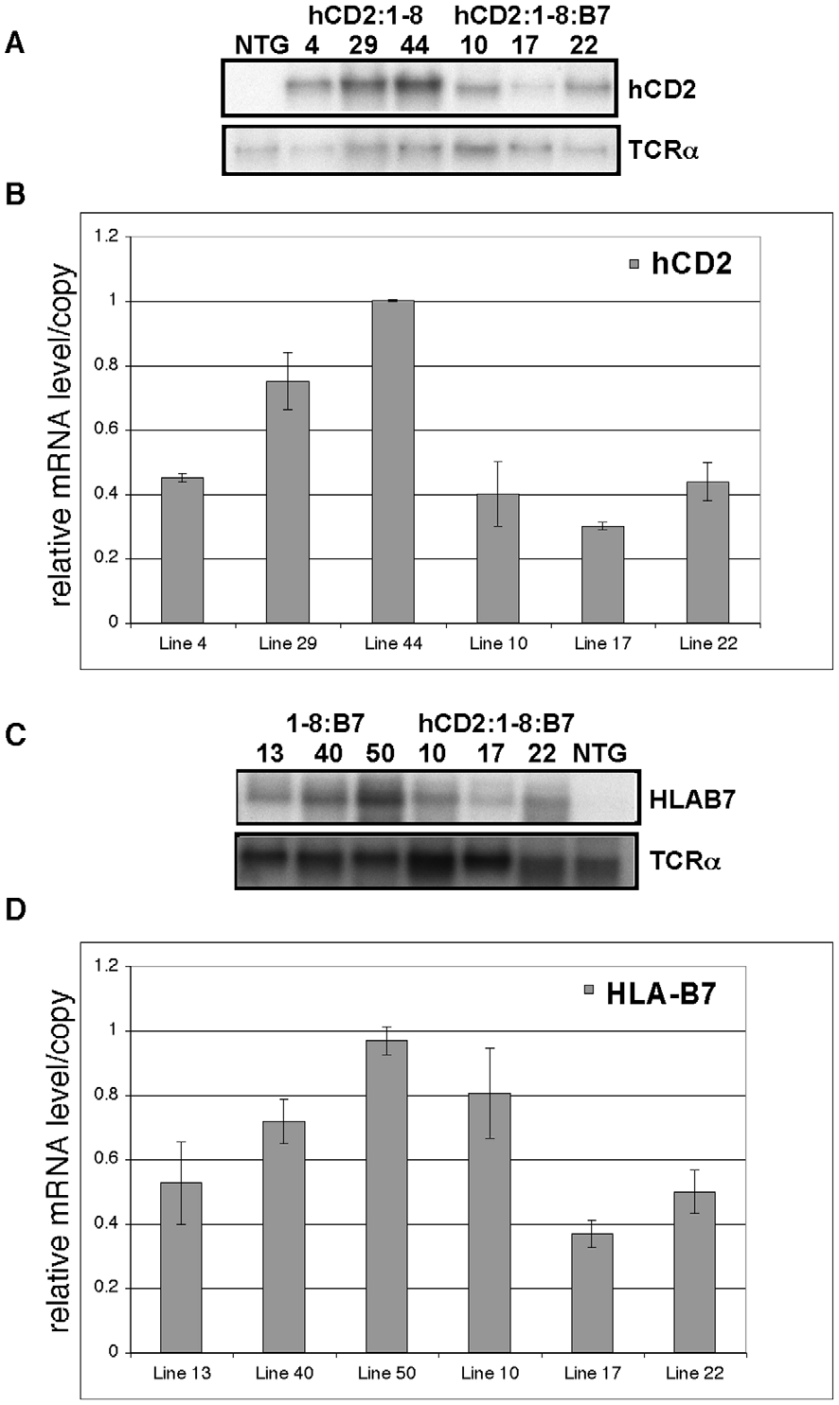
Integration site-independent expression of two reporter transgenes concurrently flanking the TCRα LCR. (**A&C**) Northern blot analyses of thymocyte mRNA from hCD2 and HLA-B7 reporter trasngenes linked concurrently (or individually) to opposite sides of the TCRα LCR. Transgenic line numbers are indicated. NTG  =  non-transgenic. (**B&D**) PhosporImager analyses of northern blot experiments. The graphs depict the mean (+/− S.E.) of three representative experiments. Y-axis values indicate the percent of the maximum transgenic hCD2 mRNA signal per copy (normalized to endogenous TCRα mRNA signal) observed in each experiment. The copy number-related (within a narrow 2–3 fold range) characteristic of full LCR activity is orientation-independent and conferred upon both reporter genes in single-reporter and dual-reporter transgene contexts.

We examined the mRNA levels produced in thymocytes from the transgene constructs described in Figure 1. PhosporImager analyses of northern blot assays indicated that both the hCD2 and HLA-B7 reporter genes were highly expressed (Fig. 2). Furthermore, reporter mRNA levels were also transgene copy number-related. In both single- and dual-reporter transgene contexts, normalized reporter transcript levels per transgene copy varied only within the narrow two- to three-fold range consistent with the integration-site independence of LCR activity [24]. These results demonstrate that the TCRα LCR can confer a major hallmark of LCR-driven gene expression, integration-site independence, upon two unrelated flanking genes at once.

**Figure 3:**
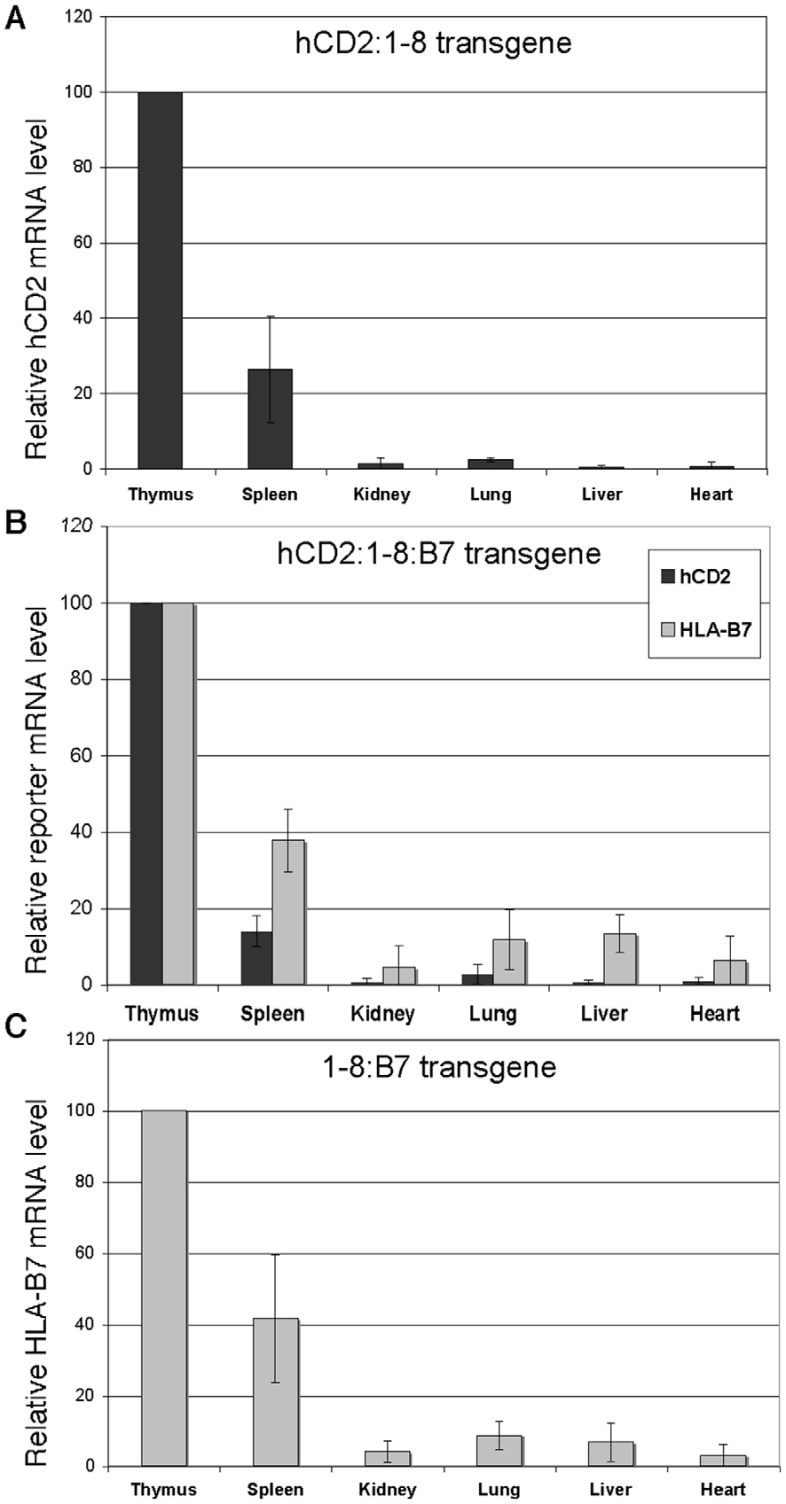
Lymphoid organs express the highest levels of both hCD2 and HLA-B7 reporter transgenes. PhosphorImager analyses of northern blots of RNA prepared from the indicated tissues of the hCD2:1-8 (**A**), hCD2:1-8:B7 (**B**) or 1:8-B7 (**C**) transgenic mice. Reporter mRNA levels are quantified and normalized to 18S rRNA signal. Y-axis values represent the mean (+/− S.D.) expression levels relative to the thymus (designated as 100%) observed among three independent lines of mice bearing the indicated transgene.

As expected, the relative tissue distribution of the upstream hCD2 reporter mRNA showed the highest levels in lymphoid organs (thymus and spleen) and very low to absent levels in other organs (Fig. 3A, 3B). Curiously, HLA-B7 transcript levels were also highest in the thymus and spleen of transgenic mice (Fig. 3B, 3C). In non-lymphoid organs, HLA-B7 reporter expression was higher (4–14%, of thymus levels) than those observed for the hCD2 reporter (0–2%). This finding would be consistent with the much wider tissue-distribution of the Dad1 gene normally found on the LCR's 3′-flank in the genome. Nevertheless, high-level expression of the endogenous Dad1 gene does not show the strong bias towards lymphoid organs displayed by the HLA-B7 reporter gene. Previous studies have shown that relative Dad1 mRNA levels seen in thymus and spleen is comparable to those seen in other organs [8]. Therefore, while the TCRα LCR is able to support high-level transcription of a 3′-flanking reporter gene that is protected from integration site-dependent position effects, it alone cannot confer upon the reporter the wide tissue-distribution of high-level activity characteristic of Dad1 gene expression.

### Placement of a second gene 3′ of the LCR suppresses ectopic expression of a 5′-LCR-flanking reporter gene in B cells

**Figure 4:**
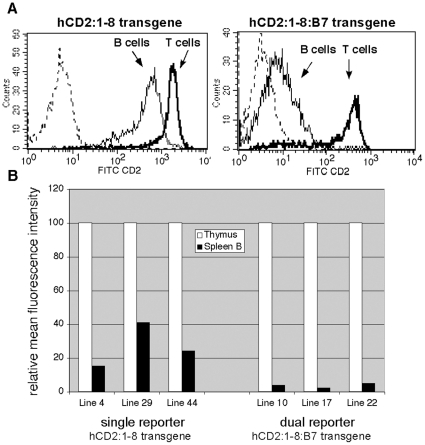
Ectopic TCRα LCR-driven upstream reporter gene (hCD2) expression is strongly suppressed by adding a second gene (HLAB7) downstream of the LCR. (**A**) Flow cytometry analyses of spleen lymphocytes from (at left) single-reporter hCD2-LCR (10 transgene copies) and (at right) two-reporter hCD2-LCR-B7 (3 transgene copies) transgenic mice. hCD2 levels are shown for spleen T cells (gated on Thy1+ cells) and spleen B cells (gated on CD19+ cells). Dashed curves indicate the non-transgenic spleen control signal. (**B**) Graph depicting mean fluorescence intensity (MFI) of hCD2 reporter protein staining in paired samples of thymocytes and isolated B cells (from the same mouse). Shown are three independent lines each of single-reporter hCD2-LCR and two reporter hCD2:1-8:B7 transgenic mice. MFI is normalized to thymocyte hCD2 expression (100%). In all cases, the substantial ectopic B cell expression of hCD2 seen from the single-reporter transgene is virtually eliminated in the presence of a second reporter gene downstream of the LCR.

The hCD2 reporter transgene is amenable to flow cytometry analyses. We therefore examined hCD2 expression levels in splenic T and B cell populations using fluorochrome-conjugated antibodies specifically recognizing the human CD2 protein (Fig. 4). As previously reported, significant levels of transgene expression (15–40% of thymus levels) are seen in spleen B cells in multiple independent lines of “single-reporter” hCD2:1-8 transgenic mice [4]. Surprisingly, levels of hCD2 protein detected in B cells from the “dual-reporter” hCD2:1-8:HLAB7 transgene were much lower (2–5% of thymus levels) than those observed from the single-reporter transgene. The hCD2 gene fragment used in both transgene constructs is identical. Furthermore, as shown in Figure 2, thymus expression levels per copy of hCD2 from both the single- and dual-reporter transgenes are comparable. Therefore we detect a selective suppression of ectopic hCD2 reporter gene expression in B cells when the HLA-B7 gene is placed downstream of the LCR.

**Figure 5:**
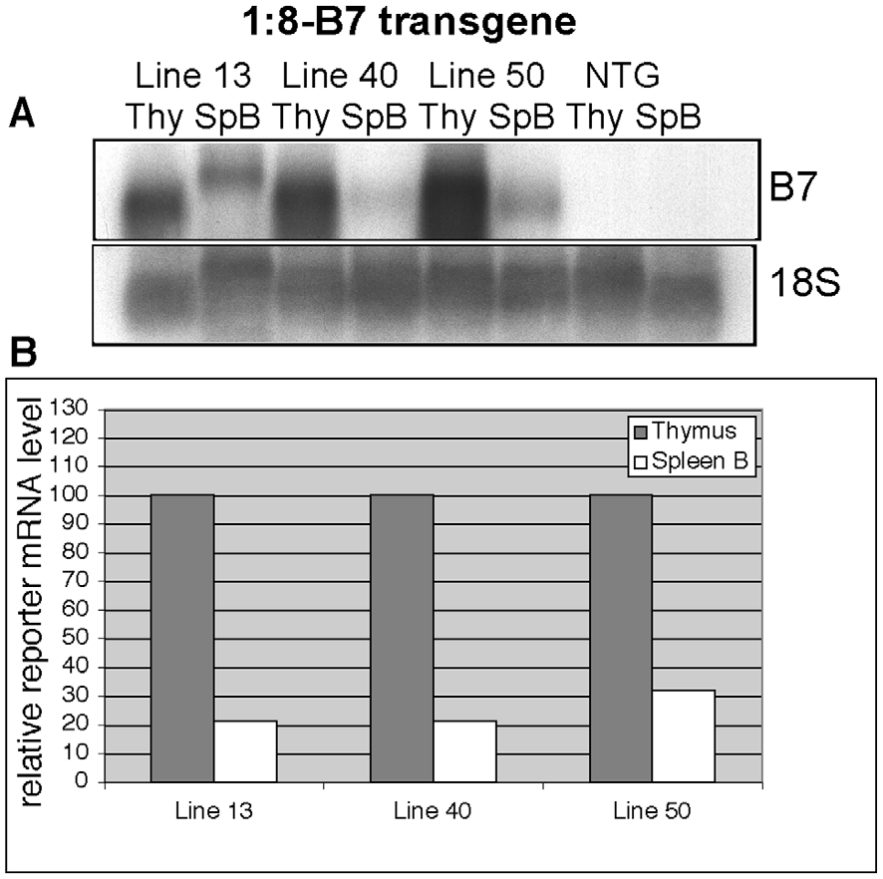
The TCRα LCR drives significant mRNA expression levels of a 3′-linked HLA-B7 reporter gene in B cells. (**A**) Northern blot analyses of RNA prepared from thymocytes and isolated spleen B cells from three independent lines of 1:8-B7 transgenic mice. Thy  =  Thymus, SpB  =  Spleen B cells, NTG  =  non-transgenic. (**B**) Graph depicting PhosporImager analyses of HLA-B7 reporter expression levels normalized to 18S rRNA. Y-axis values are expressed relative to thymus mRNA levels (designated as 100%).

**Figure 6:**
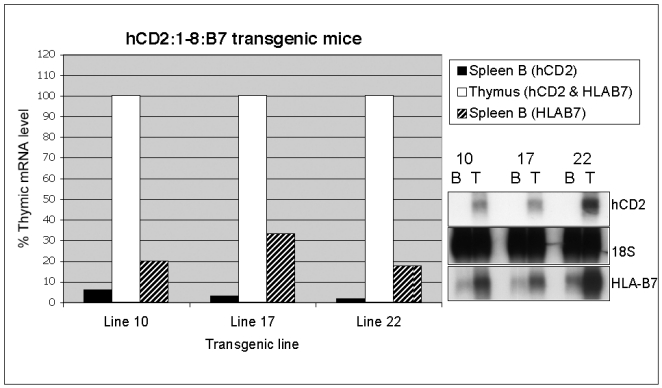
HLA-B7 reporter mRNA expression in B cells is not suppressed by the presence of a second reporter gene upstream of the TCRα LCR. PhosphorImager analyses of representative northern blot (bottom right inset) analyzing levels of spleen B cell expression of hCD2 and HLA-B7 mRNA relative to those observed in thymus (designated as 100% for all lines) in three independent lines (10, 17, 22) of hCD2:1-8:B7 transgenic mice. Reporter signals are normalized to 18S loading control signal. HLA-B7 expression levels in B cells (B), relative to thymus (T), from this dual-reporter transgene construct is similar to that seen in single-reporter 1-8:B7 transgenic mice (see Fig. 5). Levels of hCD2 mRNA from the dual-reporter hCD2:1-8:B7 transgene are as low, relative to thymus, as the hCD2 protein signals detected in flow cytometry (see Fig. 4).

The protein product of the HLA-B7 gene fragment utilized here was undetectable by flow cytometry, likely due to the absence of human β2 microglobulin in our system. Therefore, to examine expression of this reporter gene in B cells, we employed magnetic bead (MACS) technology to purify the B cell populations from transgenic mouse spleen, and prepared RNA from the isolated cells. Northern blot analyses (Fig. 5) showed that, similar to the hCD2 single-reporter transgene, significant HLA-B7 gene expression is observed in B cells of HLA-B7 single-reporter (1-8:B7) transgenic mice. Normalized reporter mRNA levels ranged from 21% to 32% of thymus levels as detected by PhosphorImager analyses. In contrast to the suppression of hCD2 reporter gene expression in B cells observed in the presence of a second reporter gene flanking the LCR, HLA-B7 mRNA levels in B cells were unchanged by the presence of the additional hCD2 reporter gene upstream of the LCR (Fig. 6). HLA-B7 transcript levels in the dual-reporter transgene ranged from 17 to 33% of thymus mRNA levels. Similar to the flow cytometry data shown in Figure 4, reporter hCD2 mRNA levels produced by the dual-reporter transgene in B cells were very low (1–6% of thymus levels).

Since HLA-B7 reporter expression in B cells is not affected by the placement of a second reporter gene into the transgene construct, these data indicate that the suppression of hCD2 reporter gene expression in B cells is not merely an artifact created by the presence of the dual reporters in the transgene. Rather, the addition of a second gene downstream of the LCR reveals a directional negative regulatory activity that limits the expression of the gene 5′ of the LCR to T cells. This is the native position of the TCRα gene locus relative to the TCRα LCR in the genome.

**Figure 7:**
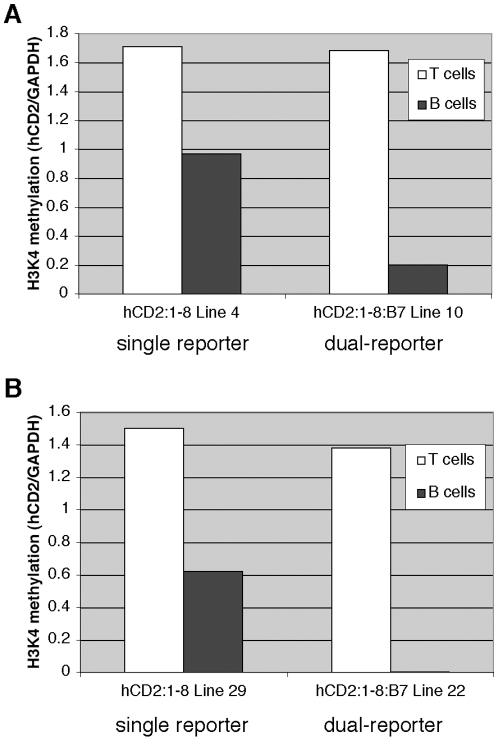
Increased T cell-selectivity of epigenetic hCD2 promoter chromatin activation in the dual-reporter transgene context. (**A**) Chromatin immunoprecipitation assay on thymocytes and isolated spleen B cells detecting the trimethyl-lysine 4 epigenetic mark on Histone H3 from single reporter (hCD2:1-8 line 4) and dual-reporter (hCD2:1-8:B7 line 10) transgenic mice. Y-axis values represent the ratio of percent H3K4me3 marks obtained at the hCD2 promoter to that detected at the endogenous GAPDH promoter, an internal standard used here as a normalizing control. (**B**) Confirmation of ChIP results using distinct, independent lines of transgenic mice bearing single reporter (hCD2:1-8 line 29) and dual reporter (hCD2:1-8:B7 line 22) transgenes. For both experiments, the Y-axis values are derived from the formula hCD2 [H3K4me3 – IgG/input]/GAPDH [H3K4me3 – IgG/input].

To corroborate, and gain further mechanistic insight into, the finding that hCD2 reporter gene expression is more T cell specific in the dual-reporter transgene context than it is in the single-reporter transgene construct, we examined the chromatin state of the hCD2 promoter region in both transgenes. We assayed for the presence of tri-methyl marks on lysine 4 of Histone H3 (H3K4me3) using chromatin immunoprecipitation (ChIP). H3K4me3 is an epigenetic histone modification associated with promoter activation and transcript initiation [25]. Figure 7 shows the percent H3K4me3 detected at the transgenic hCD2 promoter, expressed relative to that observed at the endogenous GAPDH promoter, which is used here as an internal, normalizing standard. In single-reporter transgenic mice, the fold-enrichment of H3K4me3 marks at the hCD2 promoter is only ∼1.7 to 2.4-times higher in thymocytes than that seen in spleen B cells. In sharp contrast, dual-reporter transgenic mice display greater than 8-fold higher H3K4me3 enrichment in thymocytes than the levels of enrichment observed in B cells. Thus, the levels of epigenetic chromatin activation detected at the hCD2 promoter in this system correlate with the cell type-specificity of hCD2 transcript accumulation observed. These data indicate that the hCD2 promoter region in B cells exists in different local chromatin conformations in single-and dual-reporter transgene contexts.

### A reporter gene 5′ of the TCRα LCR is not ectopically expressed in B cells in the context of a TCRα/Dad1 BAC transgene

**Figure 8:**
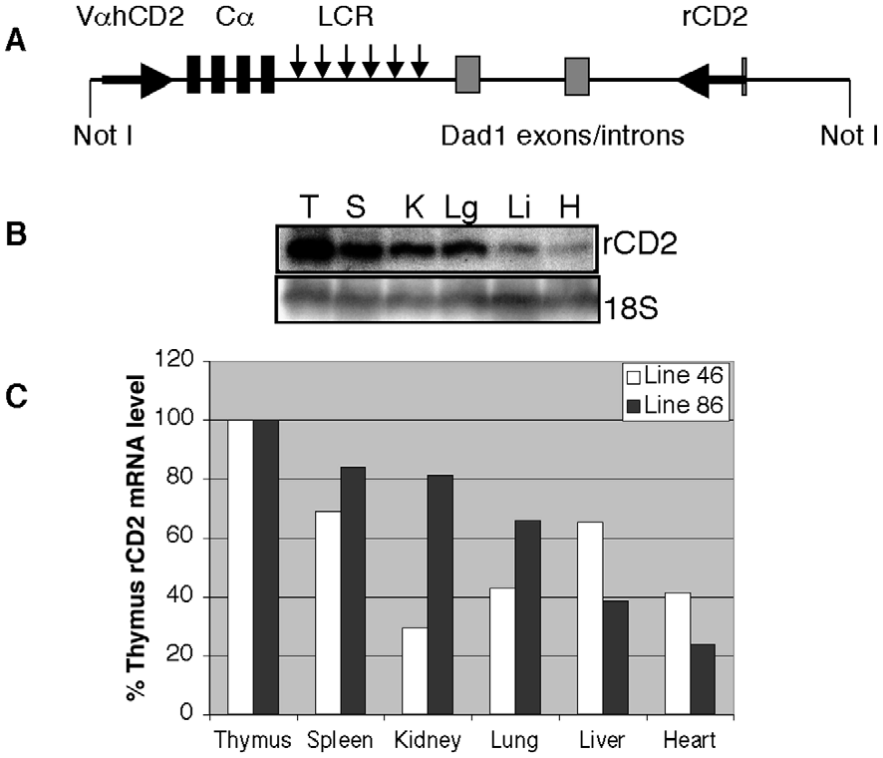
A TCRα/Dad1 reporter BAC transgene harbors an active Dad1 promoter. (**A**) Diagram of the TCRα/Dad1 dual-reporter BAC construct. VαhCD2 refers to the TCRα V-region promoter driving a human cytoplasmic tail-less human CD2 cDNA. The rCD2 refers to the BAC-resident Dad1 promoter driven rCD2 reporter cDNA. The TCRα constant region exons (Cα), HS of the TCRα LCR (small arrows) and Dad1 exons are shown. The Not I sites shown are ∼78-kb apart. (**B**) Representative northern blot analysis of rCD2 reporter expression from BAC transgenic line 86 in the indicated organs. The 18S signal is used as a loading control. (**C**) PhosphorImager analyses of northern blot experiments examining rCD2 reporter gene expression in the indicated organs of individuals representing two independent BAC transgenic mouse lines. The distribution of mRNA levels is expressed relative to the levels observed in the thymus.

Transgenes under the control of TCRα gene regulatory elements have previously been reported to be ectopically expressed in B cells [2], [3]. Based on the results of our dual-reporter transgene described above, we hypothesized that the Dad1 gene normally present 3′ of the TCRα LCR in the genome might serve to suppress such ectopic B cell expression. Therefore, we examined the expression of a TCRα V-region promoter driven hCD2 reporter cDNA placed 5′ of the TCRα LCR in the context of a bacterial artificial chromosome (BAC) containing 78-kb of DNA of the mouse TCRα/Dad1 locus. The BAC begins upstream of the Cα exons and spans the entire Dad1 gene locus including a 5.4-kb segment of non-coding DNA upstream of Dad1 Exon 1 that would contain its putative promoter region. A cDNA encoding a cytoplasmic tail-less version of hCD2 was fused in frame to the ATG translation start codon contained in a Vα11.1 promoter fragment [14], [26]. This reporter cassette was then recombined into the BAC in a position 180-bp upstream of Cα exon 1. As a reporter of Dad1 promoter function, a cDNA coding for rat CD2 (rCD2), also missing its cytoplasmic tail [27], was recombined into the BAC in frame with the Dad1 ATG start codon, thus replacing most of Dad1 exon 1. Figure 8 shows northern blot data demonstrating that rCD2 mRNA expression from the BAC is widespread and readily detectable in multiple organs. This indicates that the reporter BAC harbors an active Dad1 promoter.

**Figure 9:**
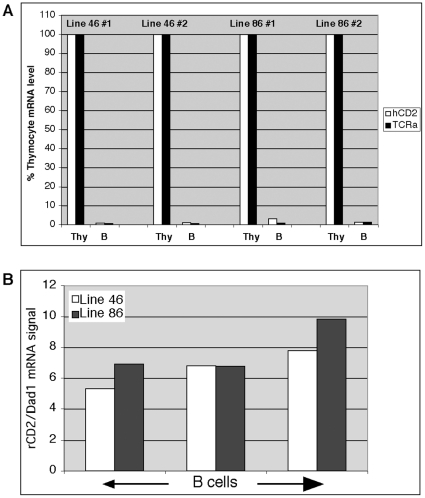
Ectopic TCRα/hCD2 reporter gene activity is absent in B cells of TCRα/Dad1 dual-reporter gene BAC transgenic mice. (**A**) Real-time reverse transcriptase-mediated PCR experiments on RNA prepared from thymocytes and isolated B cells from individual TCRα/Dad1 dual-reporter BAC transgenic mice. Y-axis values indicate the relative hCD2-Cα reporter (open bars) and endogenous TCRα (filled bars) mRNA levels within an individual mouse (thymocyte level designated as 100%). hCD2-Cα reporter mRNA signals ranged from ∼10–20% of endogenous TCRα mRNA levels. The normalizing control mRNA was β-actin. Experiments on two separate individual mice per each of two independent BAC transgenic lines are shown. (**B**) The Dad1 promoter on the BAC is active in B cells. Real time RT-PCR detection of BAC-resident, Dad1 promoter-driven rCD2 reporter expression from B cells isolated from individual BAC transgenic mice. The data are normalized to endogenous Dad1 mRNA levels.

No hCD2 protein was detectable on BAC transgenic lymphocyte cell surfaces by flow cytometry due to aberrant splicing we encountered of the hCD2 reporter (at base pair # 618 in the open reading frame of the hCD2 cDNA) to Cα exon 1 (data not shown). To circumvent this problem, we designed a real time, RT-PCR strategy to detect this BAC-specific hCD2-Cα fusion transcript. Using this quantitative assay, we examined Vα-promoter driven reporter gene activity from the BAC in thymocytes and isolated spleen B cells of BAC transgenic mice (Fig. 9A). In these experiments, B cell hCD2 reporter mRNA expression detected was only 1–3% of thymocyte levels. Similar results were obtained from simultaneous analyses of endogenous TCRα mRNA. Thus, unlike the Vα promoter-driven (single transcription unit) transgenes studied previously [2], [3], the Vα promoter-driven hCD2 reporter gene in the dual-reporter TCRα/Dad1 BAC does not display ectopic expression in B cells. Figure 9B shows RT-PCR data confirming that, as expected, the Dad1 promoter driven rCD2 reporter gene is active in B cells of the BAC transgenic mice.

## Discussion

In this report we describe two important new findings regarding the properties of the TCRα LCR and make a significant advance in our understanding of its capacity to generate T cell restricted gene expression. First, we have determined that the sequences of the LCR alone are insufficient for conferring a widespread, Dad1 like gene expression pattern upon a reporter gene linked to its 3′-end. Widely-active chromatin activating elements were previously discovered in the 3′-DNA of the LCR [16], [18], [19]. Thus, it was plausible that these elements could provide a more ubiquitous expression pattern to a 3′-linked gene than it would to a 5′-linked gene, upon which a lymphoid organ specific pattern is conferred by the full LCR [4], [16]. Although these data do not rule out a role for the LCR in Dad1 gene expression, other elements in the Dad1 locus are clearly required to generate the full widespread expression pattern characteristic of this gene.

Several LCRs, including those from the human β-like globin [28], VpreB/λ5 [29], Th2 cytokine [30] and human growth hormone [31] gene loci have been shown to regulate their multiple, related, naturally occurring target genes. We find that in T cell-bearing organs in vivo, the TCRα LCR is capable of conferring high-level integration-site independent, copy-number related gene expression upon two *heterologous* and *unrelated* genes that flank it simultaneously. It is likely that the LCR achieves this dual-gene activation function via dynamic and alternating interactions with the upstream and downstream genes. Alternating gene-LCR interactions have been observed in the β-globin multi-gene locus [28]. That we demonstrate the LCR's ability to regulate two *unrelated* flanking genes is significant because this is the LCR's natural state in the endogenous locus. It further supports the notion that elements of the LCR may be involved in regulating its natural 3′ neighbor, the Dad1 gene, despite the fact that its expression pattern is radically different from that displayed by TCRα. That the two flanking, unrelated genes can be *heterologous* (i.e. not the natural TCRα and/or Dad1 genes themselves) augurs a high degree of potential utility of the TCRα LCR in the design of vectors for genetic engineering of T cells. Several envisioned gene therapy applications require expression of multiple proteins in T cells. The most obvious of these would be the introduction of pre-rearranged TCRα and TCRβ chains that, together, would encode a receptor targeting diseased cells [32]–[34].

The question of the mechanisms generating cell type-specific patterns of lymphocyte antigen receptor gene rearrangement and expression has been the focus of much study. In general, TCR genes are activated, assembled and transcribed in T cells but not B cells. The activity of the endogenous TCRα gene is T cell restricted. A recent comprehensive study of transcription factor expression during T lineage commitment has confirmed a small group of such factors (GATA-3, TCF-1 and Bcl11b) as T lineage-specific with respect to lymphocyte development [1]. The activity of members of this group of factors may contribute to the specificity of TCRα gene expression. TCF-1 has been clearly implicated in TCRα gene expression [35]. However, TCF-1 activity has also been found to be redundant to that of LEF-1 [35], [36] which is also expressed in B cells. GATA-3 has long been suspected of involvement in TCRα enhancer (Eα) function [37]. However, subsequent studies have found GATA-3 activity to be dispensable for the function of both TCRα and TCRβ enhancers [38]. Furthermore, GATA-3 becomes differentially expressed among the classes of αβ TCR-bearing cell subsets and thus plays a role in the specialization of CD4 versus CD8 [39] and T helper-2 versus T helper- 1 cell [40] subsets. In addition, Runx and Ets family proteins have been shown to interact *in vivo* with functional DNA in the Eα/HS1 [41] and HS6 [18], [19] regions of the TCRα LCR. However, these proteins are generally not T cell restricted [1] and, in fact, are important for gene regulation in B cells [42]. Therefore, despite these important advances, a clear explanation for the restriction of TCRα gene expression to T cells has remained elusive. Nevertheless, this existing information may explain why TCRα transgenes removed from their natural genomic context [3] as well as other transcription units under the control of TCRα gene regulatory elements, such as the TCRα LCR, become expressed in both T and B cells. The latter is true whether or not transcription from the transgene construct is driven by a natural Vα promoter [2] or a heterologous promoter [4]. It is important to determine the reasons for this discordance between the endogenous TCRα locus and TCRα transgenes. The integration site-dependent position effects that transgenes can be subject to cannot explain this discrepancy. This is because ectopic B cell expression is seen from transgenes under the control of the complete TCRα LCR that, generally, are not susceptible to such position effects. Furthermore, aberrant expression of TCR chain genes in B cells appears to have deleterious consequences for B cells homeostasis *in vivo* [3]. Therefore, the further development of more physiological TCR transgenic mouse models, as well as potential TCR gene therapy applications, will require an understanding of how to restrict to T cells the TCRα mRNA generated from integrated transgene constructs.

In this report, we describe work that has revealed an important clue towards this goal. We found that the activity of the TCRα LCR, which in the genome is normally flanked by two distinct genes, is qualitatively altered by such a two-gene arrangement. The placement of a second, distinct reporter transcription unit downstream of a TCRα LCR fragment already linked to an upstream reporter gene suppresses ectopic expression of that upstream reporter gene in B cells. This unexpected inter-gene cis-regulatory interaction is observed in dual-reporter transgenic constructs employing heterologous (hCD2/HLA-B7) promoters. Prior to these findings, the most obvious explanation for the lack of strict T cell-specificity of transgenes under the control of TCRα LCR would have been the lack of specific, unidentified cis-elements of the wider endogenous TCRα/Dad1 gene locus in the transgene construction. However, our data would seem to argue against this simple hypothesis. The heterologous reporter gene system utilized here shares the “two-flanking-gene” arrangement about the TCRα LCR that is a feature of the TCRα/Dad1 gene locus. However, it is unlikely to share specific sequence elements (outside of the LCR) with the endogenous locus.

How might the findings described above apply to TCRα gene regulation? It has been shown that TCRα LCR-driven single reporter transgenes under the control of Vα [2] (as well as hCD2 and β-globin promoters [4]) become expressed in B cells. TCRα single-transgene constructs under the control of natural Vα promoters have also been shown to be ectopically expressed in B cells [3]. These prior data make clear that neither the Vα promoter, nor the full TCRα LCR can fully prevent substantial ectopic transgene expression in B cells. It is therefore of significance that we find that the Vα promoter-driven reporter gene upstream of the TCRα LCR (i.e. in the TCRα position) in our TCRα/Dad1 dual-reporter BAC transgene does not display such aberrant expression in B cells. All together, these data are consistent with the notion that the inter-gene cis-regulatory interaction observed in our heterologous dual-reporter transgene system may apply to the natural TCRα and Dad1 promoters. According to the model arising from the heterologous system, the presence of the Dad1 gene downstream of the LCR in the BAC would be playing a similar, restricting role to that observed for the HLA-B7 gene in the heterologous transgene construction. We cannot yet explicitly rule out that the HLA-B7 and Dad1 genes serendipitously share a common gene regulatory element that would be responsible for suppressing TCRα LCR activity in B cells. But this possibility seems unlikely. However, both genes do share an active promoter in our experimental systems.

In light of these results, we now hypothesize that having two distinct viable promoters flanking the TCRα LCR induces a novel promoter selection mechanism that only permits both promoters to be simultaneously active in T cells. While we cannot yet propose a particular molecular mechanism for this inter-gene cis-regulatory interaction, we can speculate from our data that such a mechanism will likely require, at a minimum, the presence of two different promoters and one or more sub-elements of the TCRα LCR that lies in between them. An example of a cis-acting regulatory activity that coordinates the function of multiple promoters in complex gene loci is that supported by the promoter targeting sequence (PTS) [43]. The first PTS was described in the Abdominal-B gene of the *Drosophila* Bithorax multi-gene complex. It has been described to have an anti-insulator function. Insulators are cis-elements that can support enhancer-blocking and/or chromatin barrier function [44]. In general, they serve to prevent functional *in cis* interactions of gene regulatory elements positioned on opposite sides of it. The anti-insulator function of the PTS would thus selectively enable an enhancer to activate a distant promoter even if an insulator exists between them.

The presence of multiple promoters and an insulator in a transcription unit enables the PTS to display a promoter targeting activity. In experiments with transgenic Drosophila embryos, this function of the PTS restricts an enhancer to a functional interaction with only one promoter when two are present in the same transgene [45]. With respect to the TCRα/Dad1 gene locus, the connection of PTS activity to insulators is intriguing. Multiple insulator activities have been described within the TCRα LCR of both enhancer blocking [46], [47] and putative chromatin barrier [19], [48] types. The DNA region containing these insulator-like activities has an apparent impact on the regulation of the TCRα locus [8], [49] and sub-elements within this DNA region are required for TCRα LCR activity *in vivo* [19], [48]. Thus, it is plausible that insulator and PTS-like activities may be cooperating to produce the differential expression patterns of reporter genes representing TCRα and Dad1 in T versus B cells when two distinct transcription units are present.

An alternative hypothesis would invoke a competition between the two distinct promoters for the regulatory activity of the TCRα LCR. The simplest explanation for any such competition would be based on relative promoter strength or position as has been hypothesized in prior studies [50]. In our transgene systems, the downstream (HLA-B7 and Dad1) promoters are normally more widely active than the upstream (hCD2 and Vα) promoters. Thus, it is also conceivable that a competition model can be based on the more ubiquitous nature of the downstream promoters used in the present study. In any case, exploring these hypotheses would be the next steps to take in understanding the coordination of the disparately regulated genes in the complex and immunologically important TCRα/Dad1 gene locus.

## Materials and Methods

This research was reviewed and approved by the Hunter College Institutional Biosafety Committee and the Hunter College Institutional Animal Care and Use Committee (protocol # BO-10/11-02)

### DNA constructs and transgenic mice

Transgenic mice were generated by pronuclear microinjection as described previously [48]. The hCD2:1-8 transgene was also previously described [4]. Briefly, a cytoplasmic tail-less hCD2 (hCD2ΔT) reporter gene [20] was linked to a 9.5-kb TCRα LCR cassette containing the DNase hypersensitive sites (HS)-1-8 [16]. Three independent mouse lines containing this construct were utilized in this study. The relative transgene copy number in the three lines (4, 29, 44) was determined to be 10, 5, and 5, respectively. The 5.5 kb EcoRV-BamHI human HLA-B7 genomic fragment (from J. Chamberlain via gift of G. Siu) previously described is devoid of the activity of its natural, cognate LCR [21]. This fragment was linked to the 3′ portion of the above-described TCRα LCR cassette in a transcriptional orientation similar to that of the Dad1 gene with respect to the LCR. Microinjection of this construct, called 1-8:B7, yielded 3 independent transgenic lines (13, 40, and 50) with copy numbers of 5, 5, and 10, respectively. The “dual-reporter” hCD2:1-8:B7 transgene is a construct that has the two above described reporter genes, hCD2ΔT and HLA-B7 simultaneously flanking the TCRα LCR. Here, hCD2ΔT was inserted 5′ of the TCRα LCR cassette and the HLA-B7 reporter is 3′ of the LCR. Three independent transgenic mouse lines bearing this construct were established (lines 10, 17, 22) with copy numbers of 3, 3, and 7, respectively.

### Bacterial artificial chromosome modification and constructs

The TCRα/Dad1 Bacterial Artificial Chromosome (BAC) reporter construct was based on BAC clone RP23-94I14 (I14) (BACPAC Resources, Oakland, CA). This BAC encompasses the TCRα/Dad1 locus ranging from about 90 kb upstream of the TCRα constant region to approximately 59 kb downstream of the Dad1 exons. BAC modification was accomplished using the Red/ET Recombination system (Genebridges, Dresden, Germany). As a reporter for the TCRα gene, a separate transcription unit (named Vα11.1hCD2ΔT) containing the Vα11.1 promoter from the pVCαK transgene [14] was inserted in frame with a cDNA encoding a cytoplasmic tail-less hCD2 protein (hCD2ΔT – a gift of M. Bevan). The 1680 bp Vα11.1hCD2ΔT fragment was inserted 180-bp upstream of the TCRα constant region in the same transcriptional orientation as TCRα. Similarly, a 703 bp rat CD2 reporter cDNA (also lacking the cytoplasmic tail) [27], was recombined into the BAC clone in frame with the ATG residing in the first exon of the Dad1 transcription unit. This also removed most of Dad1 exon 1 from the BAC. The resulting BAC construct was further modified by deleting 90-kb immediately upstream of the introduced Vα11.1hCD2ΔT fragment. This final construct named Δ5′I14h/rCD2 was digested with *Not* I to liberate the insert from the BAC vector. Because of a naturally occurring *Not* I site in the BAC insert, this digestion also removes about 17-kb between the 3′ end of the BAC vector and the Dad1 gene. The resulting fragment of 77.9-kb was purified and used to generate transgenic mouse lines. Two independent transgenic mouse lines bearing this fragment intact were generated and analyzed.

### RNA analyses

RNA from mouse organs was prepared as previously described using a single step isolation protocol [51]. Mouse tissues were dissected of fat and washed with phosphate buffered saline to minimize blood contamination then placed in RPMI containing 5% FBS until homogenized. Alternatively, purified T cells and B cells were isolated using a magnetically activated cell separation system (MACS, Miltenyi Biotec Auburn, CA) prior to RNA extraction. All MACS isolated populations were greater than 90% pure by flow cytometry analyses. Northern blot experiments were carried out using 5 µg RNA/sample run on 1% agarose gels. Samples were transferred to non-charged nylon membrane (Genescreen, Perkin Elmer, Waltham, MA) for hybridization using Quickhyb solution (Stratagene, La Jolla, CA). Human CD2 transgene mRNA was detected using a 0.5 kb *Eco* RV-*Pst* I probe from exon II of the human CD2 gene while HLA-B7 mRNA was detected with a 2 kb *Bgl* II genomic fragment. The 703-bp rCD2 cDNA was used to probe for transgenic rCD2 mRNA. To normalize for loading variation, blots were stripped and re-probed with a 0.5 kb *Sau* 3AI fragment of a TCRα constant region (Cα) cDNA or 18S rRNA probe (Ambion, Austin, TX). All probes were labeled with [α-32P]dCTP using a random primer labeling kit (Invitrogen, Carlsbad, CA). Transgene signals were normalized and quantified by PhosphorImager analyses (GE, Pittsburg, PA).

### Real-Time, reverse transcriptase-PCR analyses

Thymocytes and spleen B cells, isolated using the MACS separation technology (Miltenyi Biotech, Auburn, CA) from BAC transgenic mouse lines were analyzed by real-time, reverse transcriptase PCR. RNA was prepared from isolated cells as described above. First strand synthesis from the RNA samples was performed with the Protoscript kit (NEB). Approximately100 ng of each RNA sample was primed with oligodT primers and treated according to the manual of the kit. Typically, 1/10 of the first strand synthesis reaction was used to set up PCR reactions using the components of the HotStart kit (NEB). Real time PCR was carried out in an Applied Biosystems 7500 real time PCR system. The primers used to detect the BAC-specific reporter hCD2/Cα transcript were (forward) 5′-AGGGAACAAAGTCAGCAAGGA-3′ and (reverse) 5′-GGAGGATTCGGAGTCCCATAAC-3′. To detect endogenous TCRα mRNA, primers used were (forward) 5′-AAGATCCTCGGTCTCAGGACA-3′ and (reverse) 5′-AGCAACCTTCCTCACAAATCTG-3′. Primers used to detect the BAC-specific rCD2/Dad1 transcript were (forward) 5′-AGCTGTACCAAGGAAAGGAGCAT-3′ and (reverse) 5′- AGTTCATGACGACAAGGTGCAG-3′. Primers used to specifically detect endogenous Dad1 mRNA were (forward) 5′-GGCACTGGACTTGAGGATTCTA-3′ and (reverse) 5′-GGAAAGTAAGGGCTACAGTGAGG-3′. Primers used to detect β-actin were (forward) 5′-GAGCACAGCTTCTTTGCAGCT-3′ and (reverse) 5′-AGCCTGGATGGCTACGTACAT-3′.

### Flow Cytometry

Single cell suspensions of thymocytes and spleen cells were treated in FACS stain buffer (RPMI 1640, 3% FBS, and 10 mM HEPES buffer). 10^6^ cells in 100 µL were blocked for 20 minutes 4°C with an excess of normal mouse IgG. Cells were stained with 0.2–1.0 µg of antibody for 20 minutes at 4°C. Three washes in FACS stain buffer were done before flow cytometry analysis. In addition to analyses of thymocytes, Mouse anti-mouse CD90.2/Thy1 (Invitrogen) was used to detect splenic T cells. Rat anti-mouse CD19 (BD Pharmingen, San Jose, CA) identified splenic B cells. The appropriate population was gated, analyzed, and compared in both non-transgenic and transgenic cells for hCD2 expression using mouse anti-hCD2 (clone S5.2 - BD Pharmingen). Acquisition and analyses was done using FACScan (BD) and Cell Quest Pro software.

### Chromatin Immunoprecipitation (ChIP)

Thymocytes and B cells of analyzed transgenic mouse lines were fixed in 10 ml RPMI medium with 1% formaldehyde at room temperature for 10 minutes. Fixation was stopped by adding 600 µl of 2 M Glycine and the cells were harvested. The cells were washed twice with 1× PBS and the cell pellets were re-suspended in 1 ml 1× micrococcal nuclease (MNase) buffer (NEB) containing 2.5 µl protease inhibitor cocktail (Sigma) and 1 µl 100 mM PMSF. Nucleosomes were prepared by incubating with 500 units of MNase (NEB) for 10 minutes at 37°C. The reaction was stopped by adding 100 µl 0.5 M EGTA, pH 7.4. The chromatin was then harvested by micro-centrifugation at top speed at 4°C. The supernatant was transferred to a new tube. To 100 µl of the sheared chromatin was successively added: 1 µl protease inhibitor cocktail, 20 µl ChIP buffer 1, 15 µl of protein G coupled magnetic beads (all from Active Motif), 25 µl of 10 mg/ml Salmon Sperm DNA (Invitrogen), 1 µl of 10 mg/ml BSA (NEB), 36 µl deionized water and 3 µl of 1 mg/ml an antibody recognizing the trimethyl-lysine 4 modification on histone H3 (anti-H3K4me3) (Abcam). As a negative isotype control, 3 µl of 1 mg/ml purified normal Rabbit IgG (Jackson Immunoresearch) was added instead of the anti-H3K4me3 antibody. ChIP reactions were allowed to proceed overnight at 4°C. After the immunoprecipitation, the steps for washing, elution of chromatin, and DNA preparation were performed as described in the manual of the ChIP-IT Express kit (Active Motif). 2 µl of each of the resulting DNA solutions were used as template in standard PCR reactions and the PCR products were analyzed by Southern blotting. Blots were hybridized with probes corresponding to each of the expected individual PCR products. Primers to detect hCD2 promoter region were (forward) 5′-GGTGCAGTCTCCAAAGAGATTACG-3′ and (reverse) 5′-CTCATCTTAGGGGTTGGTTTCCTC-3′. GAPDH promoter control primers were (forward) 5′- GGCTGCGGAAAAGTTGTTGAGGA-3′ and (reverse) 5′- CTGAGTCCTATCCTGGGAACCAT-3′.
